# Albocycline Is the Main Bioactive Antifungal Compound Produced by *Streptomyces* sp. OR6 against *Verticillium dahliae*

**DOI:** 10.3390/plants12203612

**Published:** 2023-10-18

**Authors:** Carla Calvo-Peña, Rebeca Cobos, José María Sánchez-López, Ana Ibañez, Juan José R. Coque

**Affiliations:** 1 Instituto de Investigación de la Viña y el Vino, Escuela de Ingeniería Agraria, Universidad de León, 24009 León, Spain; ccalp@unileon.es (C.C.-P.); aibas@unileon.es (A.I.); 2 Biomar Microbial Technologies, Armunia, 24009 León, Spain; jm.sanchez@biomarmt.com

**Keywords:** *Verticillium dahliae*, Verticillium wilt, olive, biocontrol, antifungal, albocycline

## Abstract

Verticillium wilt is a soil-borne fungal disease that affects olive trees (*Olea europaea*) and poses a serious threat to their cultivation. The causal agent of this disease is *Verticillium dahliae*, a pathogen that is difficult to control with conventional methods. Therefore, there is a need to explore alternative strategies for the management of Verticillium wilt. In this study, we aimed to isolate and characterize actinobacteria from the rhizosphere of olive trees that could act as potential biocontrol agents against *V. dahliae*. We selected a *Streptomyces* sp. OR6 strain based on its in vitro antifungal activity and its ability to suppress the pathogen growth in soil samples. We identified the main active compound produced by this strain as albocycline, a macrolide polyketide with known antibacterial properties and some antifungal activity. Albocycline was able to efficiently suppress the germination of conidiospores. To our knowledge, this is the first report of albocycline as an effective agent against *V. dahliae*. Our results suggest that *Streptomyces* sp. OR6, or other albocycline-producing strains, could be used as a promising tool for the biological control of Verticillium wilt.

## 1. Introduction

Verticillium is a genus of ascomycete fungi that cause vascular wilt diseases in various plant hosts. These fungi can persist in the soil for up to 14 years by producing resting structures such as microsclerotia, chlamydospores, or resting mycelia [1]. Verticillium wilt is one of the most destructive fungal diseases worldwide, affecting many economically important crops such as alfalfa, almond, pistachio, peach [2,3], cotton, lettuce, potato, strawberry, ornamental plants [1], hops [4], and olive [3,5], among others. Although different species of Verticillium cause wilt, *Verticillium dahliae* is the most widespread and virulent, due to its broad host range and geographic distribution.

Olive (*Olea europaea* L.) is one of the earliest domesticated and cultivated tree species, and it has a significant historical, social, and economic value in the Mediterranean Basin as the main source of olive oil, which is a key component of the Mediterranean diet [5]. However, olive production is threatened by Verticillium wilt, a soil-borne fungal disease caused by *V. dahliae*, which has become a severe problem for olive growers in recent years [6]. Spain is the world’s leading producer of olive oil, accounting for more than half of the global production [7]. Most of the Spanish olive oil (83%) is produced in Andalucía, a region in southern Spain, where the average disease incidence has reached 20.4% [8]. However, this figure may be underestimated due to the lack of updated data [9].

Olive Verticillium wilt (OVW) is a devastating disease that has no effective control measure available. Therefore, an integrated control strategy is the most feasible approach to manage this disease. An integrated control strategy would involve pre-cultivation measures, such as strict hygiene practices during plant propagation in nurseries, heat treatment for the sanitation of olive plants, planting in disease-free soils, proper water treatment and management to prevent the spread of infective propagules, the use of organic amendments, or the development of new control methods based on novel fungicides or biological control agents. Moreover, the development of selection and breeding programs for plant material with higher intrinsic resistance to the disease would also be beneficial [5,10]. However, the lack of effective fungicides to eradicate the pathogen from the plant vascular system, and the current policies in Europe to limit the use of chemical fungicides in agriculture, are encouraging the development of new strategies to control fungal pathogens, such as the use of new and effective biocontrol agents [11].

*V. dahliae* is a soil-borne fungal pathogen that infects plants through their root system. Therefore, it is important to develop biocontrol agents (BCAs) that can inhibit its growth in soil before it reaches the roots. Ideally, these BCAs should colonize the rhizosphere and form a protective barrier against the pathogen [11]. Several fungal BCAs have been tested for their potential to control olive Verticillium wilt, a disease caused by *V. dahliae*. These include *Fusarium oxisporum* [12,13], *Trichoderma asperellum* [14], *Mucor* sp., *Rhizopus* sp., *Phoma* sp. [15], and entomopathogenic fungi such as *Beauveria bassiana* and *Metarhizium brunneum* [16]. These fungi have shown some ability to reduce the inoculum density of *V. dahliae* in soil and the severity of disease symptoms in plants. They may also produce secondary metabolites with antifungal activity (AF) against *V. dahliae* [16]. In addition, a consortium of arbuscular mycorrhizal fungi (Rhizolive) has demonstrated some efficacy in alleviating the symptoms of OVW [17,18].

Several bacterial strains have also been evaluated as potential BCAs against OVW. One of them is *Pseudomonas fluorescens* PICF7, which can suppress the growth of *V. dahliae* by competing for the niche and nutrients, producing antibacterial compounds, and inducing plant resistance [19]. Other *Pseudomonas* strains, such as *Pseudomonas* sp. PIC25, *P. indica* PIC105, and *Pseudomonas* sp. PIC141, isolated from healthy olive plants (cv. Picual), have shown in vitro antagonism against *V. dahliae*, with *P. indica* PIC105 being the most effective in reducing disease symptoms in plants [20]. Moreover, some members of the Bacillales order, such as *Paenibacillus alvei* K165 [21] and *Bacillus velezensis* [22], have demonstrated their ability to decrease the severity of Verticillium wilt in plants.

*Streptomyces* spp. are a promising alternative for the biocontrol of soil-borne fungal pathogens [23]. These bacteria belong to the order Streptomycetales, which comprises about 600 species of diverse and prolific producers of antibiotics [24]. *Streptomyces* spp. have been reported to control various plant diseases, including those caused by soil-borne fungi [25,26]. They are also abundant in different types of soils, accounting for about 10% of the total soil microbiome [27]. However, no data are available on the biocontrol of OVW by *Streptomyces* spp., despite the fact that some strains have shown AF activity against *Verticillium* spp. affecting other crops (such as cotton, potato, strawberry, and tomato) [28]. Therefore, the aim of this study was to evaluate the AF activity of *Streptomyces* spp. isolated from the rhizosphere of olive trees against *V. dahliae* and their potential to inhibit the pathogen growth in test soils.

## 2. Results

### 2.1. Isolation and Selection of Culturable Streptomycetes Associated with the Olive Rhizosphere and Their Antifungal Activity

A total of 96 putative different strains were obtained from rhizosphere soil after selection according to their different morphological and cultural characteristics. A bioassay-based in vitro screening showed that 32.3% (31 out of 96) of the isolates exhibited some degree of AF activity against the fungal pathogen *V. dahliae* V937I strain.

**Figure 1:**
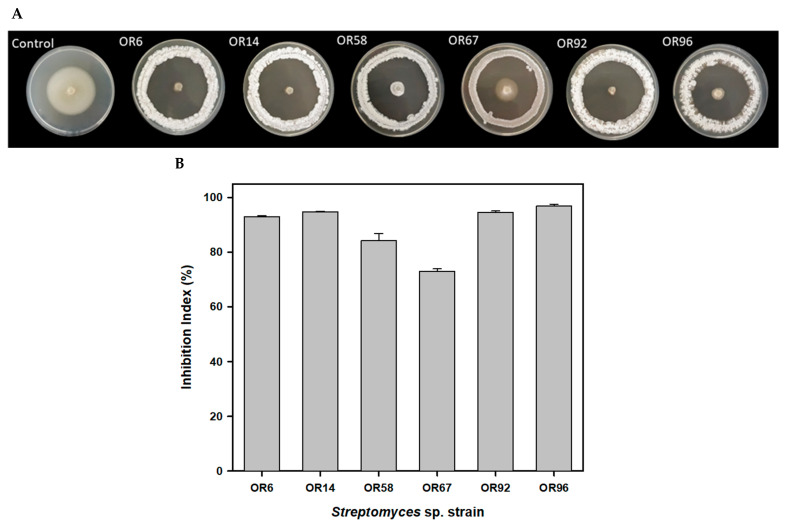
Antifungal activity of the 6 best rhizosphere isolates against the fungal phytopathogen *V. dahliae* V937I as detected by a bioassay-based in vitro screening (**A**); inhibition index of the 6 best isolates (**B**).

The six strains showing the highest Inhibition Index (I index), ranging from 73.07% for OR67 to 96.99% for OR96, were tested individually and selected for further studies (Figure 1).

### 2.2. Molecular Identification of the Selected Isolates by Partial Sequencing of 16S rRNA and Multilocus Sequence Analysis (MLSA)

**Figure 2:**
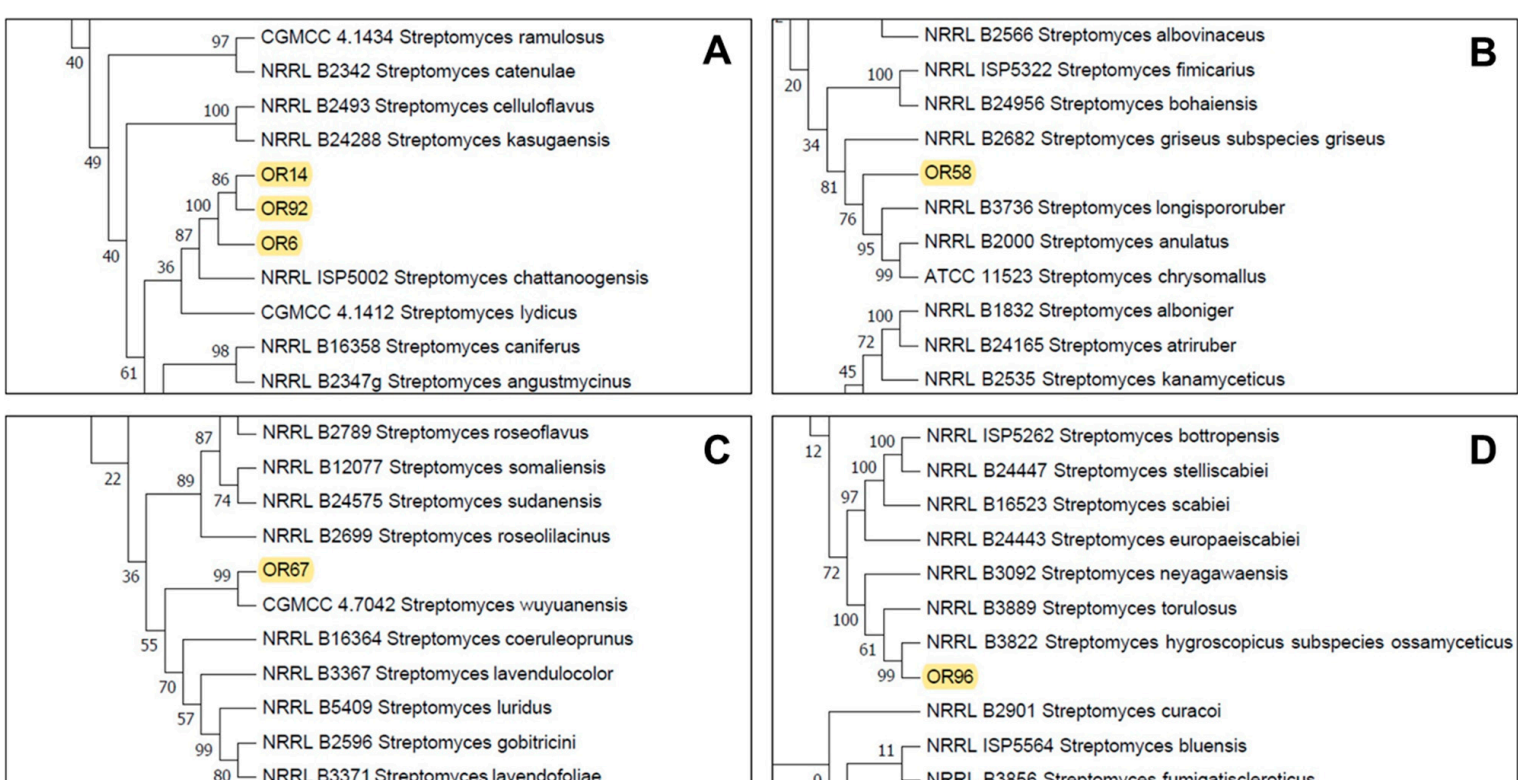
Partial details of the Neighbor-Joining tree generated from the MLSA analysis showing the location of the selected strains *Streptomyces* sp. OR14, OR92, OR6 (**A**), *Streptomyces* sp. OR58 (**B**), *Streptomyces* sp. OR67 (**C**), and *Streptomyces* sp. OR96 (**D**). See Supplementary Materials, Figure S1 for the complete tree.

The partial sequencing of 16S rRNA identified the isolates as members of the *Streptomyces* sp. genus and they were initially designated as *Streptomyces* sp. OR6, OR14, OR58, OR67, OR92, and OR96. To further characterize and accurately identify them, we performed a multilocus sequence analysis (MLSA) of five housekeeping genes (*atp*D, *gyr*B, *rec*A, *rpo*B, and *trp*B). MLSA (Supplementary Material, Figure S1) confirmed that isolates OR6, OR14, and OR92 belonged to the same species (sharing an MLSA evolutionary distance of ≤0.007; Table 1). These strains are closely related to *Streptomyces chattanoogensis* (Figure 2A), showing MLSA distances ranging between 0.043–0.045 with this species (Table 1). Strain OR58 is related to members of a subclade that included *Streptomyces longispororuber* and *Streptomyces anulatus* (Figure 2B), although its MLSA distance with these 2 species (0.013 and 0.014 respectively) clearly indicated that this isolate could represent a putative new species. Strain OR67 was placed in a subclade including *Streptomyces wuyuanensis* (Figure 2C), but its MLSA distance with this strain was 0.047, suggesting that OR67 could be a putative novel species. Similarly, strain OR96 was placed in a subclade that included *Streptomyces hygroscopicus* subsp. *ossamyceticus* (Figure 2D), but its MLSA distance with this strain was 0.018, implying that OR96 could be a putative novel species within that subclade.

### 2.3. Strain-Specific Patterns of Inhibition in Streptomyces–Streptomyces Interaction Bioassays

**Figure 3:**
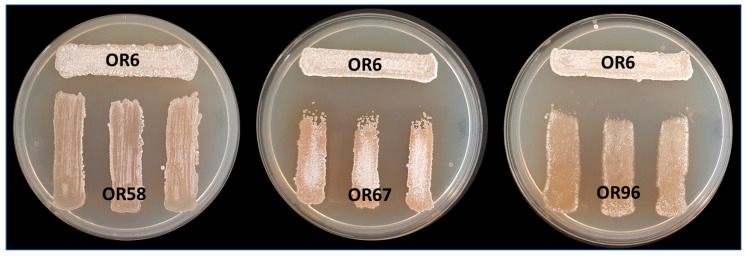
Analysis of cross interactions between the four different selected rhizosphere *Streptomyces* sp. strains after MLSA analysis. In this case, strain OR6 was acting as “tester strain” and strains OR58, OR67, and OR96 were acting as “receiver strains”. Each strain was challenged with each other on a Petri dish co-culture bioassay 3 times (*n* = 9). Similar assays were carried out using strains OR58, OR67, and OR96 as receivers.

To examine the potential negative interactions among the selected *Streptomyces* sp. strains, we performed co-culture bioassays to evaluate the effects of four strains (OR6, OR58, OR67, and OR96) on each other. Based on the MLSA analysis, strains OR6, OR14, and OR92 belonged to the same species, so we chose strain OR6 from these strains for further studies. The main goal of this analysis was to detect any possible competition or other types of negative interactions between the selected strains as a preliminary step for designing a mixture of isolates that could have higher AF activity than each individual strain in the mixture. We observed that strain OR6 inhibited the growth of the other strains to some extent (Figure 3), with inhibition rates ranging from 27.33 (±2.83)% with OR67 to 20.00 (±2.24)% with OR96, and 10.44 (±0.88)% with OR58 (Table 2).

When we used OR58 and OR67 as tester strains, they showed inhibition indexes that were always ≤8.00%, indicating low levels of inhibition. In contrast, strain OR96 did not exhibit any noticeable signs of growth inhibition or negative interactions with the other three strains in the test (Table 2).

### 2.4. Analysis of AF Activity Due to Volatile Organic Compounds (VOCs)

The capability of OR6, OR58, OR67, and OR96 to inhibit the growth of *V. dahliae* due to the putative production of VOCs was tested by using a classic double-dish chamber test. In all the cases, the AF activity due to VOC production was very low with an inhibition index of 20.54% ± 11.43 (OR6), 11.11% ± 8.57 (OR58), and 23.25% ± 6.39 (OR96), whereas no AF activity mediated by VOCs was detected for OR67.

### 2.5. Analysis of the Antifungal Activity of the Selected Strains in Small-Scale Soil Tests

**Figure 4:**
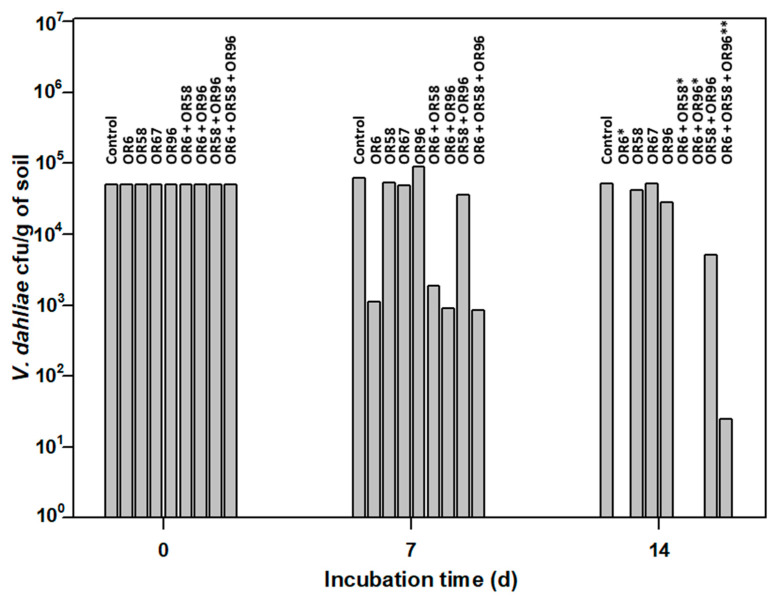
Small-scale in vitro soil assay to test the antifungal activity of the selected strains OR6, OR58, OR67, and OR96 against *V. dahliae*. * No viable *V. dahliae* was detected at 14 days (d) of the experiment. ** An average of 25 ± 3 Colony Forming Units (cfu) of *V. dahliae* per g of soil were detected at 14 d of the experiment. The values shown are the mean of two independent experiments performed in triplicate.

Many microbial isolates show good AF activity in in vitro assays, but it is unclear whether they can maintain the same activity when inoculated into soils. Therefore, we designed a small-scale in vitro assay to test the AF activity of the selected strains OR6, OR58, OR67, and OR96 against the pathogen under soil conditions. We inoculated these strains into sterile soil and measured their effects on the pathogen growth and survival (Figure 4). Sterile soil was used in this kind of experiment in order to avoid putative interferences by other microorganisms presents in the original soil sample. In this way we could be sure that the inhibitory effect observed on the pathogen was exerted by the microorganism(s) used in each individual assay.

The soil used In this study had a loamy texture, as per the USDA standard classification. The pH of the soil was 7.98, and it had an organic matter content of 6.59%. The total nitrogen content was 0.32%. These were the most characteristic aspects of the soil, and no other relevant data were available.

The pathogen was viable in this assay, as the average number of cfu/g of soil detected after 14 days of incubation was 51,500, close to the 50,000 cfu/g of soil added at the beginning of the experiment.

As shown in Figure 4, OR6 exhibited strong AF activity. After 7 days of incubation, only 1100 cfu/g of soil of the pathogen were detected, and its efficacy at 14 days was close to 100%, as less than 10 cfu/g of soil were detected in each replicate. In contrast, OR67 had no effect on the pathogen number at 7 and 14 days of incubation. Isolate OR58 did not affect the pathogen survival at 7 days of incubation but showed a slight reduction (16.0% on average) in pathogen viability after 14 days of incubation. Similarly, isolate OR96 did not inhibit the pathogen growth at 7 days of incubation but reduced it by 44% on average at 14 days of incubation. When we applied strain OR6 in co-culture with strains OR58, OR67, or OR96, their effectiveness did not decrease in small-scale soil tests, despite the negative interactions observed above (Table 2). After 14 days of incubation, the viability of *V. dahliae* in OR6–OR58, OR6–OR67, or OR6–OR96 co-cultures was always close to zero, as we detected less than 25 cfu/g of soil on average.

These results indicated that isolate OR6 was the most promising for controlling *V. dahliae* in soils, as its AF activity strongly affected the pathogen survival in soil. Therefore, we decided to identify the chemical compound responsible for the strong AF activity observed.

### 2.6. Identification of Albocycline as the Main Antifungal Compound Produced by OR6

**Figure 5:**
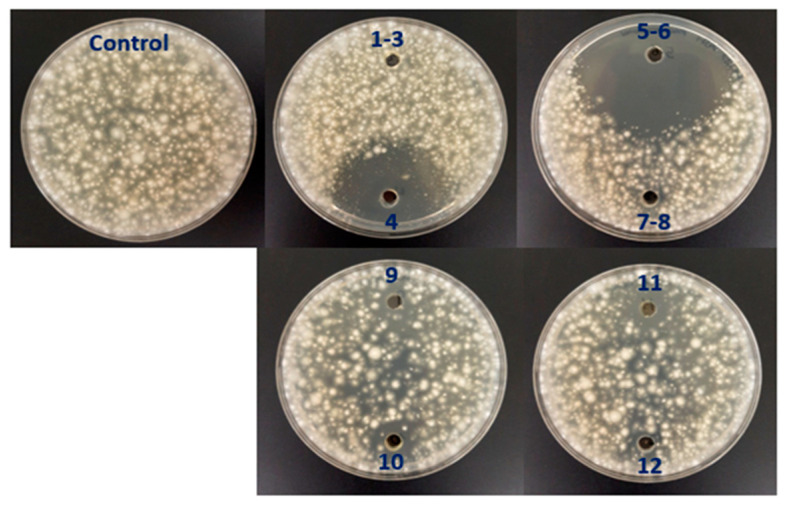
Analysis of antifungal activity against *V.dahliae* in the 12 fractions obtained by vacuum flash chromatography (VFC) during the fractionation of a crude extract (fermentation broth) obtained from a liquid culture of *Streptomyces* sp. OR6. Note that most of the total antifungal activity is concentrated in fractions 4 and 5–6.

We cultivated strain OR6 in ISP2 medium in buffled Erlenmeyer flasks for 72 h at 28 °C and 200 rpm to achieve optimal AF activity in liquid medium [30]. Next, we performed an activity-guided vacuum flash chromatography (VFC) on the crude extract (fermentation broth). Thus, after further filtration and evaporation of the solvents under vacuum, 2.28 g of extract were obtained. A 2 mg sample of this extract was analyzed by LC/MS and checked for the presence of AF activity by plate bioassay. After VFC, 12 fractions were obtained and AF activity was tested by performing a plate bioassay. Fractions 4 and 5–6 showed the highest AF activity (Figure 5).

**Figure 6:**
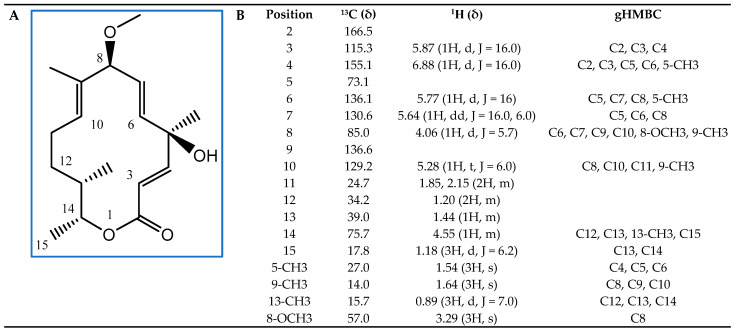
Structural elucidation of albocycline as the main antifungal compound produced by isolate OR6. (**A**) Chemical structure with carbon positions labeled; (**B**) ^13^C, ^1^H, and Heteronuclear Multiple Bond Connectivity (gHMBC) NMR spectral data of albocycline (δ (ppm), J_HH_ (Hz); CDCl_3_).

Next, we subjected these active fractions to liquid chromatography–mass spectrometry (LC/MS) and detected a major compound (Supplementary Materials, Figure S2). We determined its structure by nuclear magnetic resonance (NMR) spectroscopy (Figure 6). This analysis allowed us to identify the compound with AF activity as the 14-membered macrolide albocycline (also known as cineromycin-β-methyl ester or ingramycin). Previous studies have reported that albocycline inhibits the nicotinate [31] and peptidoglycan biosynthesis in prokaryotic microorganisms [32], whereas it also inhibits prolyl endopeptidases in eukaryotic cells [33], and exhibits both antibacterial and AF activity against various microorganisms [31,32,33,34,35,36,37,38]. Albocycline ESI-TOF-MS analysis showed an ion peak at m/z 309.2207 [M + H]^+^ (Supplementary Materials, Figure S2). The molecular formula was determined as C_18_H_28_O_4_ based on the HR ESI-TOF-MS and NMR spectral data. Extensive NMR experiments (^1^H NMR, ^13^C NMR, ^1^H-^1^H COSY, gHSQC, and gHMBC) indicated that albocycline is a 14-membered macrolide (Figure 6A). The spectroscopic data are in line with those reported in the literature [39,40]. ^1^H-NMR and ^13^C-NMR data are shown in Figure 6B.

### 2.7. Effect of Albocycline on the Germination of V. dahliae Conidiospores and Sclerotia and Calculation of MIC Value

**Figure 7:**
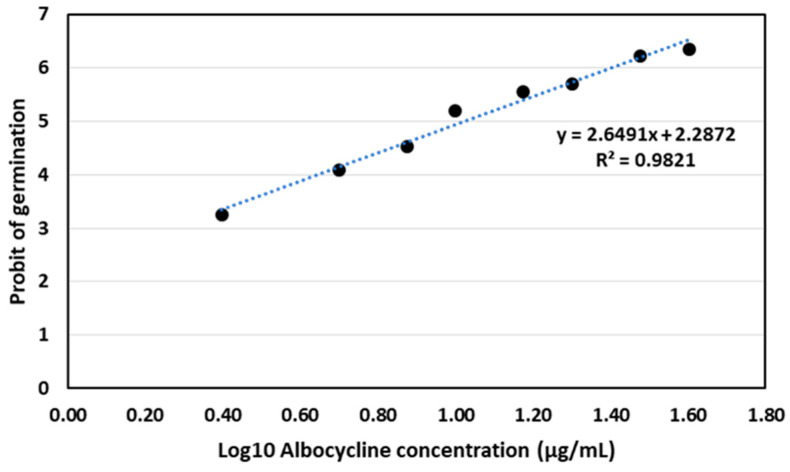
Probit analysis for germination of conidiospores of *V. dahliae* V937I strain after treatment with albocycline. The data represented are the average of 3 independent experiments made by duplicate.

Albocycline was found to be effective in inhibiting the germination of conidiospores of *V. dahliae* V937I in the tested range of concentrations of 2.5–40 μg/mL. The Probit analysis of the germination data yielded a regression curve of y = 2.6491x + 2.2872 (R^2^ = 0.9821), from which we calculated the LC50 of albocycline to be 10.57 µg/mL (Figure 7).

However, albocycline did not affect the germination of *V. dahliae* sclerotia at concentrations between 2.5 and 40 µg/mL. Microscopic observation revealed hyphal development from the germinated sclerotia, but the subsequent mycelial growth was inhibited by the AF activity of albocycline used in the assays carried out. The MIC value was calculated by standard procedures and determined to be between 5–6 µg.

## 3. Discussion

The productivity of agriculture worldwide is threatened by numerous fungal pathogenic pests that affect all crops [41,42,43]. Soil-borne fungal pathogens are particularly damaging and worrying because they spend much of their life cycle in the soil, where they can remain viable for long periods of time due to the production of different forms of resistance [5,44]. Once in contact with the sensitive plant system, they can infect it by penetrating through the root system itself and exerting their pathological activity. In many cases, by the time the symptoms of the disease are visible, the possibilities for control are practically non-existent [11]. Therefore, it is necessary to look for alternatives that allow for their control and propagation in the soil to reduce either their virulence or degree of infectivity.

Traditionally, the control of phytopathogenic fungi has been developed through the use of chemically synthesized pesticides. However, in the most advanced countries, such as those of the European Union, the medium-term policy is to ban their use and replace them with natural control methods that are non-toxic to humans and animals and are respectful of the environment. In this context, the development of BCAs emerges as a powerful tool for the control of soil-borne fungal pathogens. Although numerous scientific studies have analyzed the possible role of BCAs for the control of soil-borne fungal pathogens, relatively few have identified the substance(s) responsible for the observed AF activity.

The genus *Streptomyces* has broad biotechnological potential and is a promising candidate for the biocontrol of phytopathogenic microorganisms. The efficacy of some species of this genus in plant protection and their continued presence in the intensely competitive rhizosphere is due to their great potential to produce a wide variety of soluble bioactive secondary metabolites, including up to 8700 different antibiotics produced by actinomycetes [45], and volatile organic compounds. The capacity of *Streptomyces* as a BCA is based on different mechanisms such as induction resistance or priming plants, nutrient competition, hyperparasitism, or antibiosis [46,47].

Unfortunately, the potential effectiveness of a biological control agent (BCA) in the field is often limited by the fact that its ability to produce an antimicrobial compound in vitro does not necessarily correlate with its in situ antagonism [46]. In our study, we demonstrate that the potent antifungal activity of isolate OR6 is not only exerted in vitro but can also be detected in soil assays. This finding overcomes the aforementioned limitation, at least in soils with characteristics similar to those of the soil used in our assay. However, other isolates (OR58, OR67, and OR96) that exhibited good antifungal activity in vitro were ineffective in controlling the fungal pathogen in the soil assays we carried out. This data suggests that these isolates may not be well adapted to the characteristics of the soil used in our tests or may be unable to produce antifungal compounds when grown in soil. It is also possible that the soil contains substances or chemical compounds that inhibit antifungal production. We previously tested the AF activity of the OR6 isolate due to the production of VOCs and found it to be very limited. Therefore, it is highly probable that the potent AF activity detected in the soil test is due to the production of diffusible compounds.

Although a large number of reports dealing with Streptomyces isolates selected as BCAs to manage fungal phytopathogens can be found in the scientific literature, studies that identify the main compound(s) with AF activity are scarce. The identification of a chemical compound responsible for the AF properties of a particular strain is of great interest in order to develop formulations with a broad spectrum of antimicrobial action, promote plant growth, increase shelf life, and suppress diseases under field conditions. These characteristics would be highly attractive for technological exploitation and commercialization [47]. In our case, we were able to conclude that the AF activity of *Streptomyces* sp. OR6 was mostly due to an AF compound identified as albocycline.

Albocycline is a 14-membered macrolactone (macrolide) that is naturally produced by several *Streptomyces* strains. It was first isolated by Tanabe Seiyaku Company and The Upjohn company in 1967 and initially named as ingramycin [48]. From a structural point of view, its exact structure was elucidated by X-ray crystallography by Thomas and Chidester in 1982 [40]. Interestingly, albocycline is a 14-membered macrolactone with four stereogenic centers and three alkenes, in such a way that its skeleton is different from those of other representative 14-membered macrolide antibiotics such as erythromycin and oleandomycin. Moreover, albocycline lacks any carbohydrate moiety [49].

The production capacity of albocycline has been detected in several Streptomyces strains, including *S. maizeus* [32], *S. brunneogriseus*, *S. roseocinereu*s, *S. roseochromogenes* var. *albocyclini* [50], and *Streptomyces* sp. AR10, a strain phylogenetically close to *S. lanatus* [37]. Albocycline production has also been reported by a *Streptomyces* sp. isolated from pady soil and for an *S. sparsus* strain isolated from deep-sea sediment samples from the Bay of Bengal [36]. Albocycline production has also been detected in other actinobacteria such as *Propionicimonas* sp. ENT-18 [35]. Its production capacity is expanded with the albocycline producer *Streptomyces* sp. OR6 that, according to the MLSA analysis carried out, is a putative new species phylogenetically close to *S. chattanoogensis*, which does not appear in the bibliography as an albocycline producer.

Initially, albocycline was characterized as a potent antibacterial compound that inhibits the in vitro growth of a variety of Gram-positive and Gram-negative bacteria [48]. It is also active against methicillin-resistant *Staphylococcus aureus* (MRSA) and is equipotent with vancomycin [38]. The antibacterial properties of albocycline are due to its capability to inhibit peptidoglycan biosynthesis [38], although the exact molecular target remains to be elucidated [32]. Albocycline is also able to inhibit nicotinate biosynthesis in *Bacillus subtilis* cells [31].

Interestingly, more recently the AF activity of albocycline against *Candida albicans* [36] and the fungal phytopathogen *Sclerotinia sclerotiorum* [35] has been reported. Albocycline has also been reported to display AF activity in vitro against the fungal phytopathogen *Rhizoctonia solani*, being able to suppress Rhizoctonia dumping-off of cucumber in infection control assays [37]. Our findings indicate that albocycline is also active against *V. dahliae*, which causes Verticillium wilt in many different crops, inhibiting both conidiospore germination and mycelial growth. This is important since it adds another important fungal phytopathogen to the list of fungi sensitive to its AF action and reinforces a possible future use in the control of these pathologies.

Interestingly, it has been reported that a low concentration of albocycline (1.6 μg/sclerotia) was able to inhibit the sclerotia germination in *S. sclerotiorum* [35]. In addition, Zucchi and colleagues had previously reported that an ethyl acetate extract of *Propionicimonas* sp. ENT-18 was able to induce severe histological abnormalities in the sclerotia of *S. sclerotiorum*, affecting both the cell structure of the medullae and rind cell wall [34]. Although albocycline was identified as the main AF compound of this ethyl acetate extract, it cannot be concluded that albocycline is the cause of the observed abnormalities in the sclerotia structure [35]. In our case, albocycline was unable to inhibit sclerotia germination in the tested concentration range. This different behavior of albocycline in relation to its ability to inhibit sclerotia germination observed in *V. dahliae* and *S. sclerotiorum* could be due to structural or compositional differences in the wall of both sclerotia.

Unfortunately, there is no clear evidence about how albocycline can exert its AF activity, although we do know that it is a potent inhibitor of human prolyl endopeptidases, enzymes that are involved in the degradation of neuronal peptides containing proline residues [33]. Interestingly, albocycline lacks toxicity to mice and humans [31,32,51], which is a positive aspect that must be taken into account in a possible future application in the field.

Finally, we would like to emphasize that semi-synthetic albocycline analogs can be obtained by functionalization at three specific sites: the C2-C3 enone, the tertiary carbinol at C4, and the allylic C16 methyl group. One of the semi-synthetic C4 ester analogs obtained was twice as potent as albocycline, although only its antibacterial activity was tested [52]. Additionally, it has been reported that albocycline can be biomodified by *Streptomyces venezuelae*. However, the modification carried out consisted of a reduction in albocycline to yield a 2,3-dihydroalbocycline derivative that was antimicrobially inactive [53]. Both types of studies are very interesting since they allow us to visualize that it is possible to modify the structure of albocycline to generate similar compounds, either by chemical modification or biotransformation, that could result in new derivatives with greater AF activity.

## 4. Materials and Methods

### 4.1. Isolation of Culturable Streptomycetes Strains from the Rhizosphere of Olive Trees

Root-adjacent soil (rhizosphere) samples were collected from three adult olive trees that exhibited visual symptoms compatible with Verticillium wilt in a plot belonging to the company Río Lacarón in La Garrovilla, Spain, at 215 m above sea level (38°55′08.1″ N 6°31′27.7″ W). A visual inspection indicated that approximately 13% of the trees in that plot exhibited Verticillium wilt visual symptoms. Sampling of rhizosphere soil was made after digging to access the root system of the plants. After leaving the root system exposed, rhizosphere soil in direct physical contact with the roots was collected with a small sterile spatula. Soil samples were transferred to sterile Falcon tubes (50 mL), kept in an icebox, and preserved at 4 °C until processing. We isolated culturable actinobacteria from 1 g of soil samples by suspending them in 10 mL of Solution I (0.5% SDS; 6.0% yeast extract), homogenizing them using a vortex, and incubating them at 40 °C for 15 min. We made tenfold serial dilutions in sterile water and inoculated 0.1-mL aliquots of each dilution on starch-casein agar (SCA) [54] and International Streptomyces Project 2 (ISP2) [30] agar media, supplemented with 100 μg/mL pimaricin and 50 μg/mL nalidixic acid (Sigma-Aldrich, St. Louis, MO, USA) to prevent fungal and Gram-negative bacterial growth, respectively. We incubated the plates at 30 °C for 5 to 7 days. We selected different isolates based on their morphological and cultural characteristics, such as colony properties, presence/absence of aerial mycelia, spore mass color, distinctive reverse colony color, and production of diffusible pigments. We routinely cultivated and maintained the isolates on MEY (Maltose Yeast Extract) medium [55] at 4 °C. We maintained spore-producing isolates as spore suspensions at −20 °C in glycerol (40%).

### 4.2. Preliminary Identification of Strains by 16S rRNA Sequencing

Those strains exhibiting higher AF activity were identified by partial sequencing of 16S rRNA. Briefly, genomic DNA extraction was performed as described by Hopwood et al. [56]. The 16S rRNA genes were amplified using the oligonucleotides 27F and 1492R [57]. Isolates were identified by comparing them to corresponding sequences of the type strain found on the EzTaxon-e database [58]. (http://www.ezbiocloud.net/eztaxon/identify (accessed on 9 January 2023)). Sequence alignments were performed using the MEGA v7.0 software (http://www.megasoftware.net/ (accessed on 10 January 2023)). Evolutionary distance was calculated using the Kimura two-parameter (K2P) model for nucleotide sequences [59].

### 4.3. Identification by Multilocus Sequence Analysis (MLSA)

MLSA analyses were carried out by using five housekeeping genes: *atp*D (ATP synthase F1, β-subunit), *gyr*B (DNA gyrase B subunit), *rec*A (recombinase A), *rpo*B (RNA polymerase, β-subunit), and *trp*B (tryptophan synthase, β-subunit) [29]. We amplified the housekeeping genes using the primers and conditions described by Guo [60] and Rong [61]. The GenBank accession numbers of DNA sequences from the housekeeping genes are listed in Table 3. A phylogenetic tree was constructed from a concatenation of the five housekeeping genes. All the sequences were concatenated by joining them head to tail. DNA sequences were manually trimmed at the same position before being aligned using MEGA 7.0 software with sequences from type strains obtained from the ARS Microbial Genomic Sequence Database server (https://data.nal.usda.gov/dataset/ars-microbial-genomic-sequence-database-server (accessed on 19 January 2023)). The phylogenetic tree was constructed using the maximum likelihood method with the Kimura two-parameter model [59]. MLSA evolutionary distances were calculated using MEGA 7.0 by calculation of the K2P distance. Strain pairs having ≤0.007 MLSA evolutionary distance were considered conspecific based on the guideline empirically determined by Rong and Huang in 2012 [29].

### 4.4. In Vitro Selection of Isolates by Their AF Activity in Plate Assays

We tested all the selected isolates for AF activity using an in vitro AF assay as described previously [62]. Briefly, we inoculated the isolates on potato dextrose agar (PDA) plates in a 1.0 cm^2^ area (four isolates per plate, 1 cm from the edge of the plates). An agar plug (0.5 cm) containing *V. dahliae* V937I strain [63] was deposited in the center of the plates. Then, they were incubated for up to 12 days at 25 °C and we measured the growth inhibition zones. In order to quantify the AF activity of the best isolates, they were individually tested on MEY agar plates forming a circle at 1 cm from the edge of the plate. An agar plug containing *V. dahliae* was deposited in the center of the plate (Figure 1A). Plates were incubated at 25 °C up to 10 days. The inhibition index (I index) was calculated as follows: I index (%) = [(Rc − R)/Rc] × 100, where R is the radius of the fungal colony in the presence of the bacterial colony, and Rc is the maximum radius of the fungal colony (control). We performed this assay in triplicate for each actinobacteria tested.

### 4.5. Analysis of Antifungal Activity of Volatile Organic Compounds (VOCs)

The production of VOCs with putative AF activity was tested by using the double-dish chamber test [64]. Briefly, two sterile bottom dishes of Petri dish (9 cm in diameter) were used. One dish contained Potato Dextrose Agar (PDA) medium (Sigma-Aldrich) that was inoculated with a 9 mm diameter agar plug containing *V. dahliae*. Another dish contained a *Streptomyces* sp. culture grown in MEY agar medium [55] at 30 °C until a good sporulation was obtained (normally 3–5 days) depending on the strain tested. The two plates were faced so that the plate inoculated with the fungus was placed inverted on the plate inoculated with the bacteria. Both dishes were sealed with Parafilm and incubated at 25 °C for 14 days. Then, the inhibition index was calculated as indicated above.

### 4.6. Analysis of AF Activity in Liquid Culture Media and in Fractions Obtained during Albocycline Purification Process

We grew 100-mL liquid cultures of the OR6 strain in four different media: ISP2 [30], SPG [65], TSB, and yeast extract malt extract (YEME) [56]. We used indented 500 mL Erlenmeyer flasks containing 100 mL of liquid media. Each flask was inoculated with 10 agar plugs (0.5 cm diameter) of *Streptomyces* sp. OR6 grown on SCA plates until they produced a good amount of spores. To determine the best media for AF production, as well as the growth rate and the time of maximum AF activity, we took samples of 1 mL of culture every 24 h. The samples were centrifuged to remove cells and we tested the supernatant for AF activity using an in vitro AF plate assay against *V. dahliae* V937I strain [63] on PDA agar plates containing 0.8% of agar (*w*/*v*). We embedded 5.0 × 10^4^ fungal spores/mL in the agar media before plating. Then, we placed culture supernatant samples (60 µL) in a well on the agar plate at 1 cm from the edge of the plate. We incubated the plates at 25 °C for 4 days and measured the inhibition halos.

Plate assays to detect the presence of AF activity in the different fractions obtained during the albocycline purification protocol (see Section 4.11) were conducted as described above. Fractions (60 μL) were deposited in a well (5 mm diameter) made in the center of PDA plates containing 0.8% agar.

### 4.7. Confrontation of Rhizosphere Streptomyces sp. Isolates with Each Other

In order to rule out the possible existence of any negative interaction, the influence of the six selected *Streptomyces* sp. isolates upon each other was tested pair-wise in a bioassay following the protocol described by Schrey et al. [66]. *Streptomyces* liquid cultures were grown for three days in ISP2 medium. From the tester strain, 40 μL of this suspension culture were applied on the lower part of an SCA Petri dish, forming a line. After sporulation of the tester strain began, three parallel lines of the receiver strains were applied perpendicularly to the tester line. For each *Streptomyces* sp. pair, three tester and nine receiver lines were applied. The impact of the tester strain on the growth and formation of the receiver strain’s substrate mycelium and sporulation was recorded at the time point of the onset of sporulation in the control cultures.

### 4.8. Analysis of AF Activity in Small-Scale Soil Tests

To evaluate the AF activity in soil, we conducted small-scale tests in 50 mL Falcon tubes containing 5 g of sterile soil. Spores of each particular strain tested were obtained by growing in MEY agar plates at 28 °C and up to 6–8 days. Spores were recovered in glycerol (20%) and later were quantified by performing tenfold serial dilutions and plating on MEY plates. We inoculated the soil with 500 µL of a bacterial suspension supplemented with starch (1%) and casein (0.5%) to achieve a final concentration of 10^7^ cfu/g of soil. We homogenized the soil samples by vortexing and manually with a spatula to obtain a uniform distribution of the inoculum. We incubated the tubes at 30 °C for 48 h. Then, we added 10^5^ spores/g of soil of *V. dahliae* to each tube and homogenized them again as before. We also included a non-inoculated negative control and a positive control inoculated only with the fungal pathogen in the study. We incubated the tubes at 25 °C. Next, we collected samples of 100 mg of soil at 7 and 14 days after the bacterial inoculation and made tenfold serial dilutions in sterile water. We inoculated 0.1 mL aliquots of each dilution on PDA plates supplemented with 250 μg/mL of chloramphenicol to quantify fungal growth, and on SCA plates supplemented with 100 μg/mL pimaricin and 50 μg/mL nalidixic acid to quantify the presence of the tested actinobacteria.

### 4.9. Soil Physicochemical Properties

Physicochemical properties of the soil used for the small-scale soil test were determined in the Laboratory of Instrumental Techniques (University of León, León, Spain). Soil samples were taken at 20–30 cm deep, after removing the 5 cm of superficial soil. Five samples were taken and combined for analysis. Soil texture and mechanical analysis were performed according to the standard sedimentation method with particle size distribution in a combination of sieving and sedimentation techniques (ISO 11277:2020). The organic carbon was determined by Walkley–Black wet oxidation. The pH and EC were measured in the supernatants of soil: water 1:2.5 suspensions following 25 min of shaking and 5 min of soil settlement, and using, respectively, a micro-pH 2001 pH meter and a conductivity meter 522. Soil nitrogen was determined by the Kjeldahl method [67], which determines all soil nitrogen except that in nitrates [68]. Soil phosphorus available for vines was determined by ultraviolet–visible spectroscopy after treatment with the Olsen–Watanabe extractant. Exchangeable potassium was determined by extraction with successive aliquots of 1 M ammonium acetate (NH_4_C_2_H_3_O_2_) followed by determination by atomic absorption spectrometry (AAS; Unicam SOLAAR 969). The micronutrients (Fe, Cu, Mn, and Zn) were determined using atomic absorption spectrometry (AAS) in an air-acetylene flame [69]. Magnesium was analyzed with the CaCl_2_ method [70] determined by AAS. Available boron was measured according to the standard NF X31-122, and humus content according to the ISO 14235:1999. The total carbonates and active carbonates were determined with the Bernard calcimeter method, after extraction with ammonium oxalate 0.2 N for the last ones.

### 4.10. Obtaining Crude Extracts and HPLC Analysis

We obtained crude extracts of the OR6 strain in the selected media for antifungals production as described by Das et al. [71], with minor modifications. We mixed the cell-free culture supernatant vigorously with ethyl acetate in a 1:1 (*v*/*v*) ratio for 30 min and separated the organic layer. We evaporated the ethyl acetate extract in a vacuum concentrator CentriVap (Labconco, Kansas City, MO, USA) and dissolved the residue in 80% methanol to a final concentration of 1 mg/mL. We tested samples of 60 µL of the crude extract for antifungal activity using the antifungal bioassay described above. We filtered the rest of the sample by 0.45 µm pore Corning^®^ Costar^®^ Spin-X^®^ centrifuge tube filters (Merck KGaA, Darmstadt, Germany) and stored them at −20 °C until analysis. We analyzed the crude extracts by high performance liquid chromatography (HPLC) following the chromatographic method described by Awla et al. [72] using an Agilent 1200 Series Gradient HPLC System (Agilent Technologies, Santa Clara, CA, USA) equipped with a quaternary pump delivery system (G1311A), a preparative autosampler (G1329A), a diode array multi-wavelength detector (G7115A), and an analytical fraction collector (G1364F) equipped with an Autosampler Thermostat (G1330B). We injected samples of 10 µL and resolved them on an analytical Lichospher RP18 column (40 × 250 mm; 5 µm) (Teknokroma, San Cugat del Vallés, Spain).

### 4.11. Purification of Albocycline by Vacuum Flash Chromatography (VFC)

Albocycline was purified from a crude extract (fermentation broth) by vacuum flash chromatography (VFC) as follows. *Streptomyces* sp. OR6 was grown in 500-mL indented Erlenmeyer flasks containing 125 mL of ISP2 medium. Each flask was inoculated with 10 agar plugs (0.5 cm diameter) of the bacterial strain grown on SCA plates until a good sporulation had been obtained. Cultures were incubated at 30 °C and 150 rpm for 72 h. Fermentation broth (6 L) was shaken with the adsorption resin Amberlite XAD-1180 (Dupont, Mississauga, ON, Canada), and filtered using Radifil RW50 as filtration coadjuvant (Agrovin, Alcázar de San Juan, Spain). The exhausted broth was discarded and the mixture of resin and mycelium was extracted with 3L of EtOAc/MeOH (3:1). After further filtration and evaporation of the solvents under vacuum, 2.28 g of extract were obtained. The extract was analyzed by LCMS, and checked for the presence of AF activity by plate bioassay.

A 2 mg sample of the obtained extract was dissolved in 400 μL of MeOH. The sample was analyzed by HPLC (1290 Infinity II, Agilent) coupled to an Agilent 6230 time-of-flight LC/MS (LC/TOF) mass spectrometer. The chromatographic column used was an Agilent Zorbax Eclipse Plus, C18 RRHD (2.1 × 50 mm; 1.8 μm particle size), gradient system MeOH/H_2_O (0.1% formic acid), 20% to 100% over 8 min, UV detection 220 nm, and flow rate 0.6 mL/min. Once checked that the obtained extract exhibited AF activity, it was subjected to VFC (Vacuum Flash Chromatography) using silica gel (40–60 μm, 60 A) (Thermo Scientific^TM^, Waltham, MA USA) as a stationary phase and for the mobile phase a gradient of Hexane/EtOAc/MeOH of increasing polarity was used to obtain a total of 12 fractions. All the fractions were analyzed by LCMS, as described above, and tested for AF activity. Active fractions eluted with Hexane-EtOAc 1:1 and 3:7 showed the presence of a major compound in their LC/MS analysis (Supplementary Materials, Figure S2).

### 4.12. Structural Elucidation of Albocycline

The molecular formula of the major compound detected in the active fractions was determined based on the HR ESI-TOF-MS and NMR spectral data. Extensive NMR experiments (^1^H NMR, ^13^C NMR, ^1^H-^1^H COSY, gHSQC, and gHMBC) were carried out in order to elucidate its structure. ^1^H-NMR and ^13^C-NMR data were recorded on a Varian “Mercury 400” spectrometer (Agilent Technologies) at 400 and 100 MHz, respectively. gHMQC and gHMBC experiments were carried out using an inverse resonance probe. Chemical shifts are reported in ppm relative to solvent (CDCl_3_ δH 7.26, δC 77.0). MS data were recorded on an Agilent 6230 time-of-flight LC/MS (LC/TOF) mass spectrometer.

### 4.13. Analysis of the Ability of Albocycline to Inhibit Conidiospore Germination

The ability of albocycline to inhibit conidiospore germination was analyzed using a modified protocol of López-Moral [73]. Conidial suspensions were obtained from 12-day-old colonies of *V. dahliae* isolate V937I growing on PDA and adjusted to 1 × 10^6^ conidia/mL. In parallel, a 5 mg/mL albocycline-concentrated solution in methanol was used to prepare more diluted 20, 40, 60, 80, 100, 120, 140, 160, 180, and 200 µg/mL solutions in sterile distilled water, in order to avoid a putative inhibitory effect of methanol on conidiospore germination. Subsequently, a 5 µL drop of the conidial suspension was placed in the center of a microscope coverslip; then, a 5 µL drop of the albocycline solution was mixed. The albocycline was evaluated at the following final concentrations: 2.5, 5, 7.5, 10, 15, 20, 30, and 40 µg/mL. A concentration of 0 µg/mL consisting of a 5 µL drop of the conidial suspension mixed with a 5 µL drop of sterile distilled water was used as a negative control. The coverslips were placed inside Petri dishes containing water agar and used as humid chambers. They were incubated at a temperature of 25 °C in the dark for a period of ten hours. After the incubation period, a 5 µL drop of 0.01% acid fuchsine in lactoglycerol (1:2:1 lactic acid:glycerol:water) was added to each coverslip to stop conidial germination. The coverslips were then mounted on a slide. The assay was performed in triplicate for each concentration and repeated twice. For each replicated coverslip, 100 randomly selected conidia were observed at a magnification of ×400 using a phase-contrast microscope (Olympus CX41), and germinated conidia were counted. Conidia were considered germinated when the germ tube was at least as long as the longitudinal axis of the conidia. The inhibition of conidial germination (RGI; %) was estimated with respect to the control using the following formula: RGI = [(G_control_ − G_Alb_)/G_control_] × 100 where G_control_ = percentage of germinated conidia after incubation in water and G_Alb_ = percentage of germinated conidia after incubation in the albocycline solution. The data obtained were plotted to obtain a regression line and an equation from which the Inhibitory Concentration of albocycline causing a 50% growth inhibition (IC50) was calculated by Probit analysis of the mortality data.

The calculation of the Minimum Inhibitory Concentration (MIC) was carried out by performing a classic disk-diffusion test [74,75] in PDA plates. The amount of albocycline per disk ranged from 5 to 50 µg.

## 5. Conclusions

A *Streptomyces* sp. OR6 strain isolated from olive rhizosphere exhibited notable AF activity in vitro against the phytopathogenic fungus *V. dahliae*. AF activity was mainly exerted by the macrolide albocycline. This strain brings together a series of very interesting properties that could in the future result in a possible commercial use as a biopesticide, such as its use in the design of biofertilizers, including: (i) the produced albocycline is active against several of the most important phytopathogenic fungi affecting many crops including *V. dahliae* (this work), *R. solani* [33], and *S. sclerotiorum* [35]; (ii) the ability to show AF activity in soil assays; (iii) albocycline is able to strongly inhibit the germination of *V. dahliae* conidiospores; (iv) since it is a strain isolated from the rhizosphere of olive trees, it is assumed to have a good adaptation to colonize that microenvironment, in which the infection of the tree by the pathogen occurs, where it can exert its AF activity; (v) finally, it should be highlighted that, as previously described in the literature, albocycline does not exhibit toxicity against mice and humans, a positive aspect that should be taken into account in its possible future field application.

## Figures and Tables

**Table 1:** Identification of the 6 rhizosphere selected isolates by MLSA analysis and MLSA distances from strains phylogenetically near to our isolates.

	MLSA (Kimura 2-Parameter) Distance *					
Strain	OR6	OR14	OR58	OR67	OR92	OR96
*Streptomyces* sp. OR6						
*Streptomyces* sp. OR14	0.002					
*Streptomyces* sp. OR58	0.130	0.130				
*Streptomyces* sp. OR67	0.113	0.112	0.095			
*Streptomyces* sp. OR92	0.004	0.002	0.132	0.114		
*Streptomyces* sp. OR96	0.111	0.111	0.114	0.099	0.112	
*Streptomyces chattanoogensis* NRRL ISP5002	0.044	0.043	0.128	0.127	0.045	0.112
*Streptomyces longispororuber* NRRL B3736	0.125	0.125	0.014	0.097	0.127	0.109
*Streptomyces anulatus* NRRL B2000	0.127	0.127	0.013	0.099	0.129	0.110
*Streptomyces wuyuanensis* CGMCC 4,7042	0.115	0.113	0.111	0.047	0.115	0.102
*Streptomyces hygroscopicus* subsp. *ossamyceticus* NRRL B3822	0.114	0.114	0.118	0.103	0.116	0.018

* Strain pairs having ≤0.007 MLSA evolutionary distance were considered conspecific based on the guideline empirically determined by Rong and Huang in 2012 [29].

**Table 2:** Inhibition index (Ii) between the 4 best rhizosphere strains selected according their antifungal activity against *V. dahliae* V937I. Each strain was challenged with each other on a Petri dish co-culture bioassay 3 times (*n* = 9).

Receiver Strain	Tester Strain							
	OR6		OR58		OR67		OR96	
	Ii (%)	SD	Ii (%)	SD	Ii (%)	SD	Ii (%)	SD
OR6			0.44	0.88	6.00	1.73	0.00	0.00
OR58	10.44	0.88			3.33	1.73	0.00	0.00
OR67	27.33	2.83	8.00	1.41			0.00	0.00
OR96	20.00	2.24	6.44	1.67	2.44	1.67		

**Table 3:** GenBank accession numbers of DNA sequences corresponding to partially sequenced genes used in the MLSA analysis of the different rhizosphere *Streptomyces* sp. strains tested.

Strain	Genbank Accession Numbers					
	16S rRNA	*atp*D	*gyr*B	*rec*A	*rpo*B	*trp*B
OR6	OR506195	OR527395	OR527401	OR527407	OR527413	OR527419
OR14	OR506196	OR527396	OR527402	OR527408	OR527414	OR527420
OR58	OR506197	OR527397	OR527403	OR527409	OR527415	OR527421
OR67	OR506198	OR527398	OR527404	OR527410	OR527416	OR527422
OR92	OR506199	OR527399	OR527405	OR527411	OR527417	OR527423
OR96	OR506200	OR527400	OR527406	OR527412	OR527418	OR527424

## Data Availability

Not applicable.

## Supplementary Materials

The following supporting information can be downloaded at: https://www.mdpi.com/article/10.3390/plants12203612/s1, Figure S1: *Streptomyces* phylogenetic tree inferred from concatenated partial sequences of the housekeeping genes (*atp*D, *gyr*B, *rec*A, *rpo*B, and *trp*B) of rhizosphere isolates (OR6, OR14, OR58, OR67, OR92, and OR96) with type strains obtained from the ARS Microbial Genomic Sequence Database server; Figure S2: Analysis of antifungal activity against *V. dahliae* in the 12 fractions obtained by vacuum flash chromatography (VFC) during the fractionation of a crude extract (fermentation broth) obtained from a liquid culture of *Streptomyces* sp. OR6.
