# Supplementary material for: Albocycline Is the Main Bioactive Antifungal Compound Produced by Streptomyces sp. OR6 against Verticillium dahliae
Source: Plants (Basel). 2023 Oct 18;12(20):3612. doi: 10.3390/plants12203612 (PMC10610244; doi:10.3390/plants12203612)

Supplementary Material. Figure S1. *Streptomyces* phylogenetic tree inferred from concatenated partial sequences of the housekeeping genes (*atp*D, gyrB, *rec*A, *rpoB,* and *trpB*) of rhizospher isolates (OR6, OR14, OR58, OR67, OR92 and OR96) with type strains obtained from the ARS Microbial Genomic Sequence Database server. The tree was constructed using the maximum-likelihood method based on Kimura two-parameters model (nucleotide-based analysis). The trees were subjected to 1000 bootstrap replications and values (expressed as percentages) greater than 50% are given in the nodes. Subclades containing the isolated strains(highlighted in bold blue font) and related type strains are shown yellow shaded. Bar scale reflects number of substitutions per site.

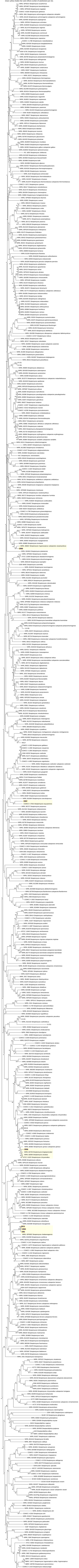

Supplement: Supplementary file 1 [file plants-12-03612-s001.zip › Supplementary Materials-Figure S1.pdf]
