# Supplementary material for: Albocycline Is the Main Bioactive Antifungal Compound Produced by Streptomyces sp. OR6 against Verticillium dahliae
Source: Plants (Basel). 2023 Oct 18;12(20):3612. doi: 10.3390/plants12203612 (PMC10610244; doi:10.3390/plants12203612)

Supplementary Material. Figure S2. (A) HPLC chromatogram corresponding to the active fraction 5-6 obtained after fractionation of a *Streptomyces* sp. OR6 crude extract by vacuum flash chromatography (VFC). Note the presence of a majority peak with a retention time of 4.81 min corresponding to a compound identified as albocycline; **(B)** Spectra obtained by High-resolution electrospray inonization time-of-flight mass spectrometry (HR ESI-TOF-MS) of albocycline peak. Molecular ion m/z 309.2008 $[M+H]^+$, 331.1820 $[M+Na]^+$, 639.3757 $[2M+Na]^+$.

A

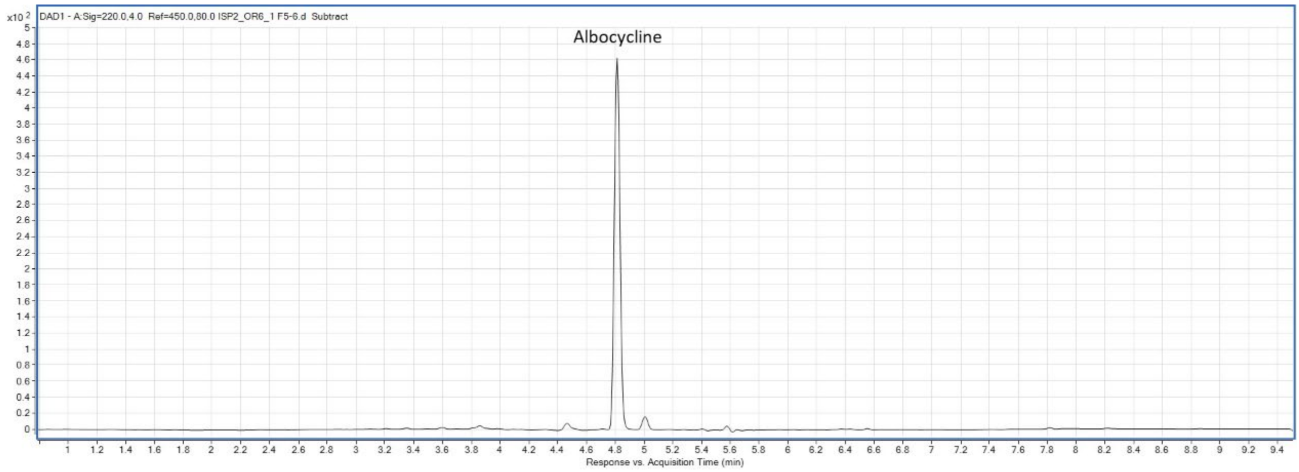

B

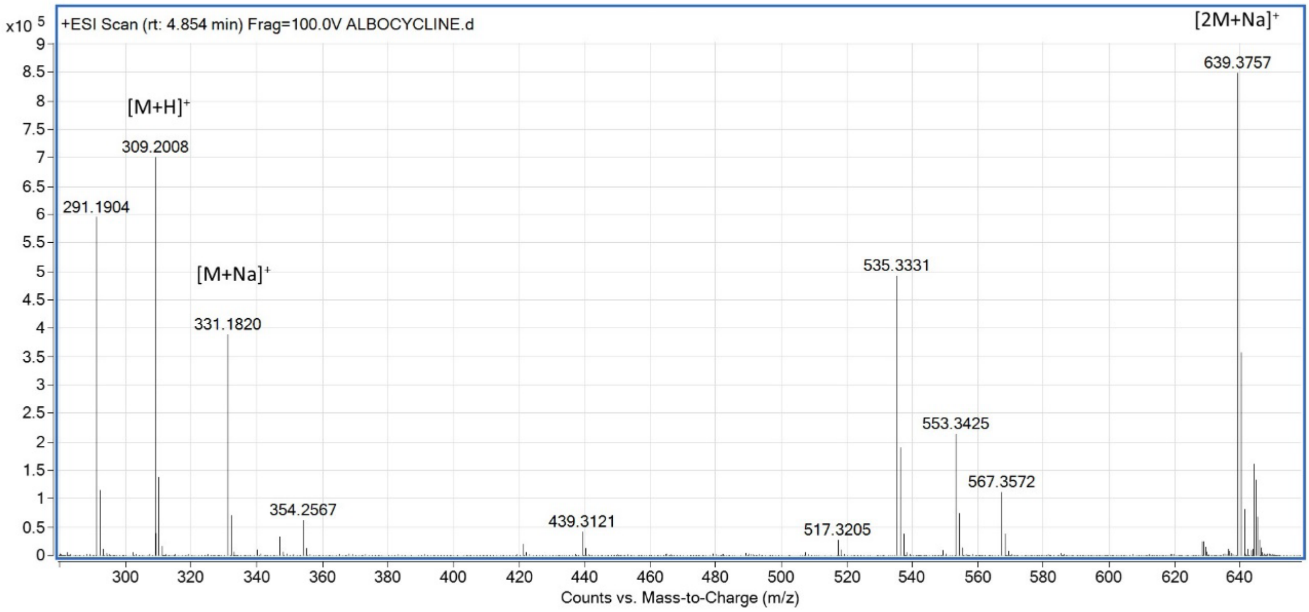

Supplement: Supplementary file 1 [file plants-12-03612-s001.zip › Supplementary Material-Fig S2.pdf]
